# Peripheral and soft-tissue vascular malformations in the 2025 ISSVA classification: a practical MRI approach

**DOI:** 10.1186/s13244-026-02475-2

**Published:** 2026-11-06

**Authors:** Shuji Nagata, Asako Kuhara, Tomoko Kugiyama, Miyuki Sawano, Shohei Mizushima, Masamichi Koganemaru, Shuichi Tanoue

**Affiliations:** 1 Department of Radiology, Kurume University School of Medicine, Kurume, Fukuoka, Japan; 2 Department of Radiology, Saitama Sekishinkai Hospital, Sayama, Saitama, Japan

**Keywords:** Vascular malformations, Magnetic resonance imaging, Magnetic resonance angiography, Arteriovenous malformations, Classification

## Abstract

**Abstract:**

The 2025 update of the International Society for the Study of Vascular Anomalies (ISSVA) classification introduced several imaging-relevant changes, including the recognition of potentially unique vascular anomalies. Although the main framework for vascular tumors remains unchanged, with continued subdivision into benign, borderline, and malignant tumors, the most radiologically relevant structural changes concern vascular malformations, which were reorganized into fast-flow lesions, slow-flow lesions, and developmental anomalies of named vessels. These updates are important for radiologists because imaging plays a central role in assessing hemodynamic characteristics, defining lesion extent, and identifying findings relevant to procedural planning and multidisciplinary management. This educational review presents an author-derived four-step framework that organizes established MRI observations for peripheral and soft-tissue vascular malformations according to the structure and terminology of the 2025 ISSVA classification, including hemodynamic assessment, vascular components, tissue composition, lesion extent, and treatment-relevant anatomy.

**Key Points:**

***Question*** Imaging-relevant organizational and terminology changes in the 2025 ISSVA classification require practical guidance for consistent MRI assessment and reporting of peripheral and soft-tissue vascular malformations.

***Findings*** An author-derived four-step, flow-based framework organizes hemodynamics, dominant vascular components, tissue composition, lesion extent, and treatment-relevant anatomy into structured MRI assessment.

***Critical relevance statement*** The MRI-focused framework provides a practical way to apply the 2025 ISSVA classification consistently in image interpretation, terminology, and reporting of peripheral and soft-tissue vascular malformations.

**Graphical Abstract:**

**Figure:**
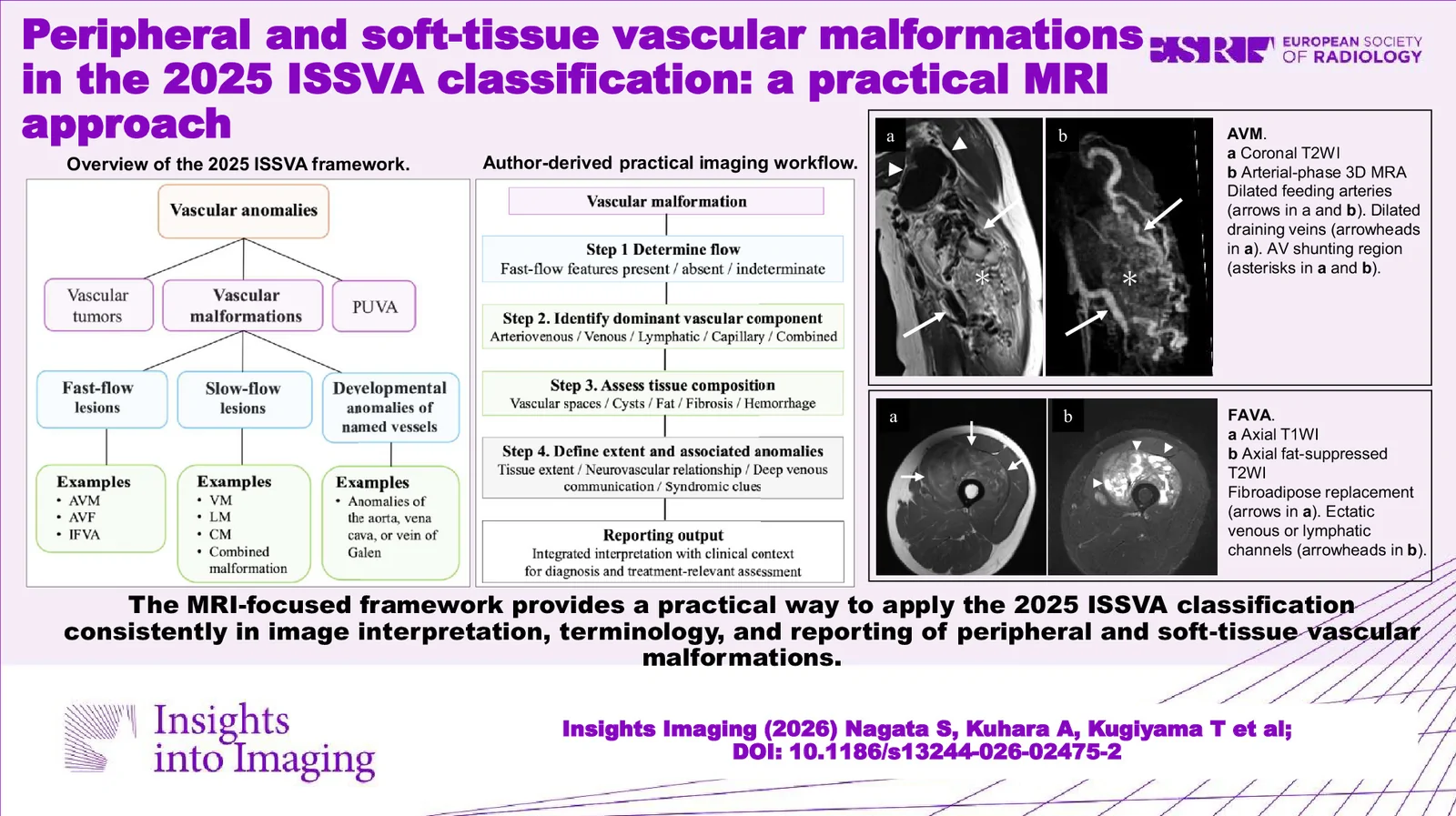

## Introduction

The 2025 update of the International Society for the Study of Vascular Anomalies (ISSVA) classification is highly relevant to radiologists, as it provides a framework to support image interpretation, diagnostic communication, and treatment planning [1–3]. The biologic distinction between vascular tumors and vascular malformations was originally emphasized by Mulliken and Glowacki and was later incorporated into the ISSVA classification framework [4–6]. Rather than serving solely as a taxonomic system, the updated classification highlights concepts that are closely related to imaging assessment, particularly flow characteristics, lesion extent, tissue composition, and involvement of named vessels [1, 2]. These imaging findings are central to distinguishing vascular tumors from vascular malformations, differentiating fast-flow from slow-flow lesions, and informing management planning, including observation, sclerotherapy, embolization, surgery, and targeted medical therapy [3, 7].

Although vascular tumors remain an important component of the ISSVA classification, the overall framework has largely remained unchanged in the 2025 update, with continued subdivision into benign, borderline, and malignant tumors. In contrast, vascular malformations underwent several structural changes of particular relevance to radiologists [1, 2]. Radiologists are often asked not only to suggest a diagnosis but also to assess flow characteristics, draining veins, and the involvement of named vessels [3, 7]. These observations can influence referral pathways, procedural planning, and multidisciplinary treatment decisions [3, 7]. Although flow assessment provides the initial branch point, final imaging characterization requires integration of vascular morphology, tissue composition, lesion extent, clinical findings, and, when available, angiographic, pathologic, and genetic information.

Importantly, many of the imaging observations relevant to vascular malformations—including assessment of flow characteristics, vascular morphology, tissue composition, and lesion extent—are established principles of vascular-anomaly imaging and were not introduced by the 2025 ISSVA update. The principal imaging-relevant contribution of the 2025 classification is instead organizational: vascular malformations are now structured primarily by flow characteristics, with separate recognition of slow-flow vascular components and developmental anomalies of named vessels, along with newly introduced or revised entities and terminology. The practical challenge for radiologists is therefore not to adopt entirely new imaging techniques, but to translate these updated classification concepts into a structured imaging assessment. To address this practical gap, we propose an author-derived four-step educational workflow that reorganizes established imaging principles to align with the structure and terminology of the 2025 ISSVA classification.

Emphasis is placed on hemodynamic characteristics, dominant vascular components, tissue composition, lesion extent, treatment-relevant anatomy, and diagnostic pitfalls. Fibroadipose vascular anomaly (FAVA) is discussed to illustrate the importance of integrating flow characteristics, vascular architecture, and tissue composition. The review primarily addresses MRI assessment of peripheral and soft-tissue vascular malformations and is not intended to provide a comprehensive organ-specific review of all vascular anomalies. Accordingly, this educational review presents a practical flow-based MRI approach to the assessment and reporting of peripheral and soft-tissue vascular malformations within the framework of the 2025 ISSVA classification.

## Materials and methods

### Literature search and review development

We conducted a narrative literature search of PubMed/MEDLINE through August 2026. Search terms included combinations of “ISSVA,” “vascular malformation,” “vascular anomaly,” “magnetic resonance imaging,” “MRI,” “arteriovenous malformation,” “venous malformation,” “lymphatic malformation,” “capillary malformation,” “fibroadipose vascular anomaly,” and “intramuscular fast-flow vascular anomaly.” Official ISSVA classification documents and glossary materials were reviewed separately. We prioritized original studies, consensus or classification documents, and imaging-focused reviews relevant to classification, MRI findings, differential diagnosis, and treatment-relevant assessment. Reference lists of selected articles were also reviewed to identify additional relevant publications. Because the purpose was educational rather than systematic evidence synthesis, formal risk-of-bias assessment and systematic-review methodology were not applied.

### Development of the imaging workflow

The four-step workflow was developed by the authors as an educational construct rather than through a formal consensus or validation process. Its structure was derived from the organizational principles of the 2025 ISSVA classification and synthesized with established imaging approaches described in the vascular-anomaly literature and the authors’ clinical experience. The steps were arranged to reflect a practical sequence for MRI interpretation: assessment of hemodynamics, identification of the dominant vascular component, characterization of tissue composition, and evaluation of lesion extent and treatment-relevant anatomy. The framework has not been prospectively tested or formally validated.

### What changed in the 2025 ISSVA classification?

**Fig. 1:**
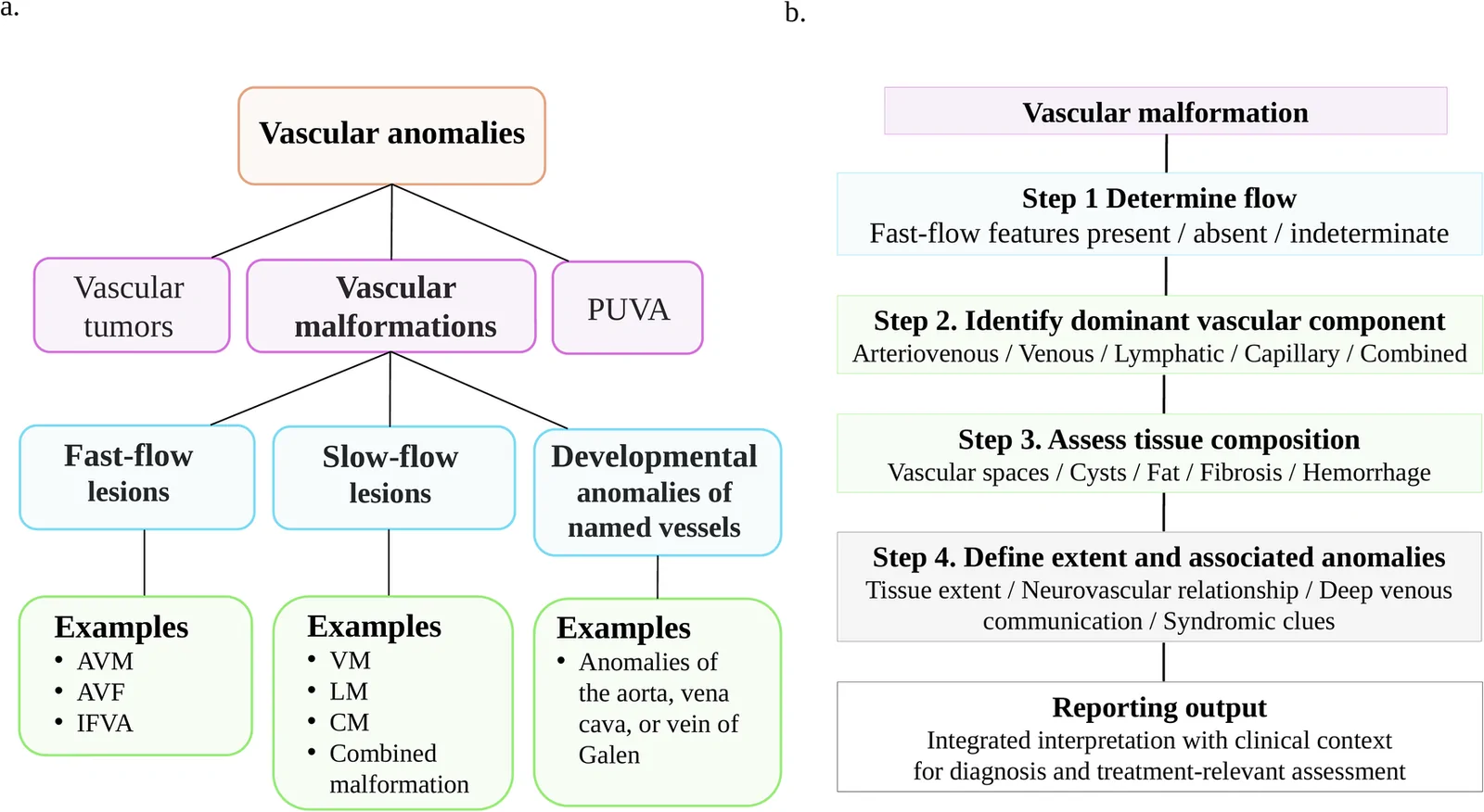
Overview of the 2025 ISSVA framework and practical imaging workflow for vascular malformations. **a** Simplified overview of the 2025 ISSVA framework. Vascular anomalies are organized into vascular tumors, vascular malformations, and potentially unique vascular anomalies (PUVA). Vascular malformations are broadly grouped into fast-flow lesions, slow-flow lesions, and developmental anomalies of named vessels. **b** Author-derived four-step educational framework for MRI assessment. Imaging assessment begins with determination of flow characteristics, followed by identification of the dominant vascular component, assessment of tissue composition, and definition of lesion extent, anatomic relationships, and treatment-relevant findings. As a practical caution, increased vascularity or early enhancement alone does not establish arteriovenous shunting. Of note, this framework is intended to organize these imaging observations and is not part of the official ISSVA classification or a validated diagnostic algorithm. AVM, arteriovenous malformation; AVF, arteriovenous fistula; IFVA, intramuscular fast-flow vascular anomaly; VM, venous malformation; LM, lymphatic malformation; CM, capillary malformation; FAVA, fibroadipose vascular anomaly; ISSVA, International Society for the Study of Vascular Anomalies

The 2025 ISSVA classification organizes vascular anomalies into vascular tumors, vascular malformations, and potentially unique vascular anomalies (PUVA) (Fig. 1a) [1]. PUVA comprises lesions that cannot be clearly assigned to either vascular tumors or vascular malformations [1, 2].

The most imaging-relevant change is the reorganization of vascular malformations into fast-flow lesions, slow-flow lesions, and developmental anomalies of named vessels [1, 2]. Slow-flow lesions are further characterized according to their capillary, lymphatic, venous, or combined components. This framework encourages radiologists to first determine flow characteristics, then identify the dominant vascular components and associated developmental vascular anomalies. The principal changes from the 2018 classification are summarized in Table 1.

**Table 1:** Imaging-relevant changes from the 2018 to the 2025 ISSVA classification

2018 ISSVA classification	2025 ISSVA classification	Imaging relevance and practical terminology
Vascular anomalies were divided primarily into vascular tumors and vascular malformations; lesions of uncertain tumor or malformation status were placed in a separate provisional list.	PUVA is recognized as a third high-level category alongside vascular tumors and vascular malformations.	Lesions with ambiguous or incompletely defined biological features need not be forced into either the tumor or malformation category. Radiologists should describe the imaging phenotype and recommend multidisciplinary correlation when classification remains uncertain.
Vascular malformations were organized as simple malformations, combined malformations, malformations of major named vessels, and malformations associated with other anomalies. CMs, LMs, VMs, and AVMs were included among simple malformations.	Vascular malformations are organized primarily into fast-flow malformations, slow-flow malformations, and developmental anomalies of named vessels. AVMs and AVFs are classified as fast-flow lesions, whereas capillary, lymphatic, venous, and combined malformations are classified as slow-flow lesions.	Flow assessment becomes the initial organizing step. Imaging should first determine whether convincing arteriovenous shunting is present and then identify the individual vascular components.
“Simple” and “combined” were overarching classification categories.	“Simple” is no longer used as a principal classification designation. Combined slow-flow malformations are defined by the coexistence of two or more slow-flow vascular components.	Reports should identify the individual vascular components and their flow characteristics rather than use a “simple malformation” label or only a broad combined malformation label.
Intramuscular capillary-type hemangioma was classified using terminology suggesting a vascular tumor.	IFVA is introduced as an isolated fast-flow vascular malformation and replaces the former term “intramuscular capillary-type hemangioma.”	IFVA should be considered in an intramuscular hypervascular lesion with fast-flow features but without the typical angioarchitecture of an AVM. Diagnosis should integrate clinical, imaging, pathologic, and, when available, molecular findings.
CMs were categorized primarily by clinical pattern and associated anomalies; low-resistance capillary malformation was not recognized as a distinct subtype.	Low-resistance CM, also termed CM with faster flow, is specifically recognized as a subtype of capillary malformation.	Increased vascularity or early enhancement should not automatically be interpreted as an AVM. A nidus, enlarged feeding arteries, and definite early venous drainage should be sought before diagnosing arteriovenous shunting.
“Malformations of major named vessels” included abnormalities involving large arteries, veins, or lymphatic vessels.	The category is reframed as “developmental anomalies of named vessels.”	Imaging should evaluate vessel origin, course, caliber, continuity, aplasia or hypoplasia, stenosis, dilatation, anomalous communication, and dependence of venous drainage on persistent embryonic vessels.

This table provides a simplified imaging-oriented comparison and does not reproduce the complete hierarchical structure of either classification

*AVF* arteriovenous fistula, *AVM* arteriovenous malformation, *CM* capillary malformation, *IFVA* intramuscular fast-flow vascular anomaly, *ISSVA* International Society for the Study of Vascular Anomalies, *LM* lymphatic malformation, *PUVA* potentially unique vascular anomaly, *VM* venous malformation

**Fig. 2:**
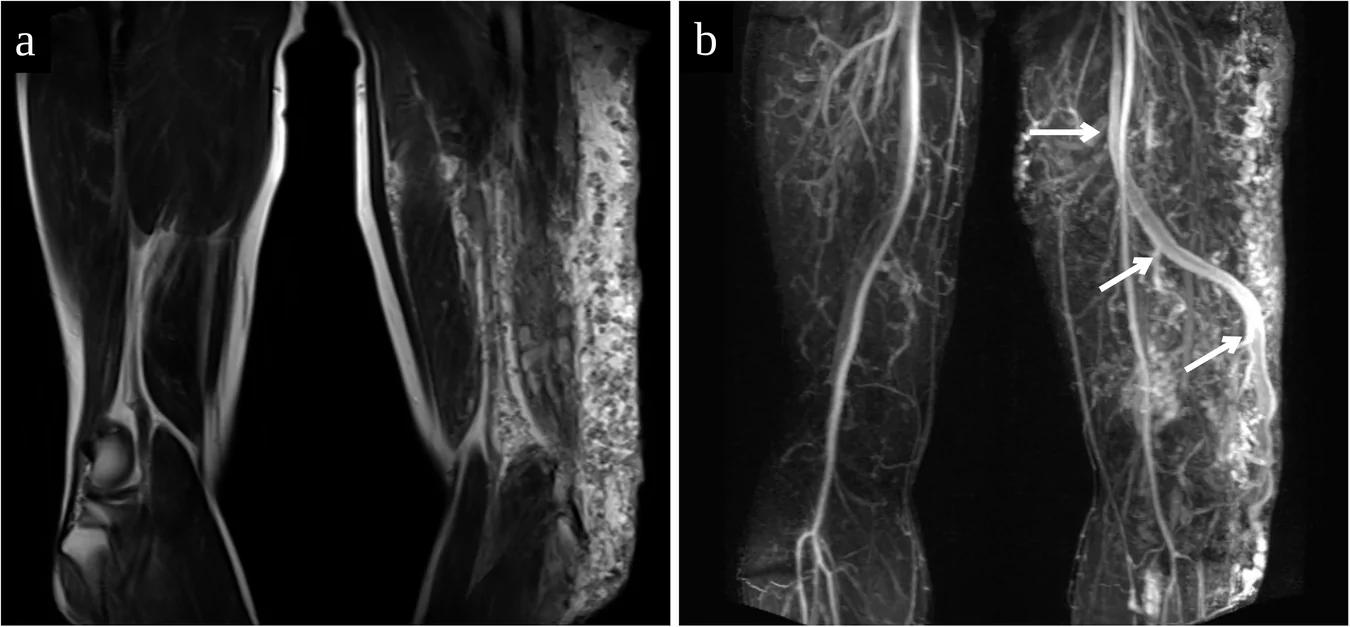
Persistent lateral marginal vein in Klippel–Trénaunay syndrome. **a** Coronal T2-weighted image shows subcutaneous soft-tissue overgrowth of the left thigh with multiple dilated and tortuous superficial venous channels. **b** Venous-phase three-dimensional MR angiography demonstrates extensive superficial venous abnormalities, including a persistent lateral marginal vein (arrows). Assessment of the extent and course of the anomalous vein is important because communication with the deep venous system and its relationship to adjacent structures may affect procedural planning. The diagnosis was based on the characteristic clinical and imaging findings

The former category of “malformations of major named vessels” has been reframed as “developmental anomalies of named vessels” [1, 2]. Radiologists should therefore assess the origin, course, caliber, continuity, stenosis, dilatation, abnormal communication, or absence of the named vessels involved, because these findings may influence diagnosis and procedural planning. A persistent lateral marginal vein in Klippel–Trénaunay syndrome is shown as a representative example (Fig. 2).

Molecular characterization has also become increasingly important in the contemporary classification of vascular anomalies. Many vascular malformations are associated with somatic or germline alterations involving the PI3K–AKT–mTOR or RAS–MAPK signaling pathways, although the specific genetic drivers vary among phenotypes. These molecular findings increasingly complement clinical and imaging assessment and may provide a rationale for targeted medical therapy in selected patients. Imaging remains important for defining the anatomic and hemodynamic phenotype, identifying appropriate lesions for further diagnostic evaluation, and monitoring treatment response [8, 9].

### Complementary roles of imaging modalities

MRI and MR angiography are central to the evaluation of peripheral and soft-tissue vascular malformations because they provide comprehensive assessment of lesion extent, tissue composition, hemodynamic characteristics, and treatment-relevant anatomy. Ultrasound with Doppler is particularly useful for the initial assessment of superficial lesions and can evaluate compressibility, vascularity, flow direction, and spectral flow characteristics. CT may be useful for detecting phleboliths, calcification, and osseous involvement, while CT angiography can provide vascular mapping in selected cases. Conventional angiography remains important for detailed assessment of feeding arteries, arteriovenous shunting, and draining veins in selected fast-flow malformations, as well as for procedural planning. The choice and combination of imaging modalities should be tailored to lesion location, suspected hemodynamics, and the clinical or procedural question.

### MRI protocol

MRI protocols should be tailored to the anatomic location, lesion size, suspected flow characteristics, and clinical purpose of the examination. The imaging field of view should include the entire lesion and its relationship to adjacent tissues and relevant named vessels. Extended or whole-limb coverage should be considered for extensive or multifocal lesions, limb overgrowth, involvement of multiple anatomic compartments, or suspected abnormalities of the deep venous system or persistent embryonic vessels.

A practical examination should combine conventional anatomic imaging to evaluate lesion extent and tissue composition with dynamic contrast-enhanced MR angiography to assess hemodynamics and vascular architecture [7, 10, 11]. Conventional MRI should include T1-weighted imaging and fluid-sensitive imaging, such as fat-suppressed T2-weighted or short tau inversion recovery imaging, preferably in at least two orthogonal planes. T1-weighted imaging is useful for assessing anatomy, fat, hemorrhage, thrombus, muscle atrophy, and fibrofatty replacement, whereas fluid-sensitive sequences help define lesion extent, venous or lymphatic spaces, septations, fluid–fluid levels, edema, and involvement of adjacent tissues. T2*-weighted imaging may provide additional information regarding low-signal foci caused by phleboliths, calcification, hemosiderin, or blood products, although radiographic or CT correlation may be required to confirm mineralization.

When hemodynamic assessment is required, time-resolved contrast-enhanced MR angiography is preferred and should be performed with sufficient temporal resolution to distinguish arterial inflow and early venous drainage while maintaining adequate spatial resolution and anatomic coverage [12]. When time-resolved imaging is unavailable, conventional contrast-enhanced three-dimensional MR angiography may be performed using appropriately timed arterial- and venous-phase acquisitions. The dynamic or multiphasic series should be assessed for feeding arteries, a nidus or fistulous connection, early venous filling, draining veins, and associated abnormalities of named vessels. Source images should be reviewed in addition to maximum-intensity-projection images, as small vascular connections may be obscured or overlap on projection images.

Postcontrast imaging may provide additional information regarding lesion extent, enhancement pattern, septal or solid components, fibrosis, and inflammatory changes. Diffusion-weighted imaging may be added when a vascular tumor, solid cellular component, or alternative soft-tissue neoplasm is included in the differential diagnosis, but diffusion findings should not be interpreted in isolation. A suggested practical MRI protocol is summarized in Table 2.

**Table 2:** MRI protocol for peripheral and soft-tissue vascular malformations

Sequence/acquisition	Main purpose	Practical considerations
Field of view/anatomic coverage	Define lesion extent and anatomic relationships	Select the field of view to include the entire lesion, adjacent anatomic structures, and relevant named vessels; consider extended or whole-limb coverage for extensive or multifocal lesions, limb overgrowth, or suspected abnormalities of the deep venous system or persistent embryonic vessels.
T1-weighted imaging	Anatomy, fat, hemorrhage, thrombus, and fibrofatty change	Acquire according to lesion location and extent; use an appropriate plane to define anatomic relationships.
Fat-suppressed T2-weighted imaging or STIR	Lesion extent, vascular or cystic spaces, septations, and edema	Acquire in at least two orthogonal planes.
T2*-weighted imaging	Phleboliths, calcification, hemosiderin, and blood products	Consider radiographic or CT correlation when mineralization is suspected.
Precontrast fat-suppressed T1-weighted imaging	Baseline for assessment of enhancement and intrinsic T1 hyperintensity	Useful for identifying hemorrhage, proteinaceous material, or thrombus before contrast administration.
Time-resolved contrast-enhanced MRA	Hemodynamic assessment and detection of arteriovenous shunting	Preferred when hemodynamic assessment is required. Use sufficient temporal resolution to distinguish arterial inflow from early venous drainage while maintaining adequate spatial resolution and anatomic coverage; review source images in addition to MIP images.
Conventional multiphasic contrast-enhanced MRA	Alternative assessment of vascular anatomy and hemodynamics when time-resolved MRA is unavailable	Obtain appropriately timed arterial- and venous-phase acquisitions and assess feeding arteries, early venous filling, draining veins, and associated named-vessel abnormalities.
Delayed postcontrast imaging	Delayed or progressive enhancement, lesion extent, and internal components	Particularly useful for slow-flow lesions, including venous malformations, and for evaluating septal, solid, fibrous, or inflammatory components.
Diffusion-weighted imaging	Assessment of a solid cellular component or alternative soft-tissue neoplasm	Optional; consider when a vascular tumor or other solid soft-tissue lesion is included in the differential diagnosis, and interpret together with conventional MRI findings.

DWI may be added when a vascular tumor or another solid soft-tissue lesion is included in the differential diagnosis

*FS* fat-suppressed, *MIP* maximum-intensity projection, *MRA* MR angiography, *STIR* short tau inversion recovery, *T1WI* T1-weighted imaging, *T2WI* T2-weighted imaging, T2*WI T2*-weighted imaging

### A practical imaging workflow for vascular malformations

Based on the organizational principles of the 2025 ISSVA classification and established imaging literature, we propose a practical four-step imaging approach for radiologists (Fig. 1b) [1–3, 7, 10, 13, 14]:

(1) Is there convincing evidence of fast flow or arteriovenous shunting? (2) Which vascular component is dominant? (3) Is the lesion composed predominantly of vascular spaces, cysts, fat, fibrosis, hemorrhage, or a combination of these? (4) What is the full lesion extent, and which anatomic findings may affect treatment or procedural risk?

A structured final report can integrate flow characteristics, dominant vascular components, tissue composition, lesion extent, named-vessel abnormalities, and treatment-relevant anatomy.

### Step 1: Determine flow characteristics

The first step is to determine whether the lesion shows convincing fast-flow features, no convincing fast-flow features, or indeterminate hemodynamics. Imaging findings suggestive of fast-flow malformations include prominent flow voids on spin-echo MRI, enlarged arterial feeders, nidus-like vascular structures, early arterial enhancement, early venous filling, and enlarged draining veins on dynamic contrast-enhanced T1-weighted imaging or MR angiography [7, 10]. Increased vascularity or early enhancement alone should not be considered sufficient evidence of arteriovenous shunting; these findings should be interpreted together with enlarged feeding arteries, a nidus or fistulous connection, early venous filling, and enlarged draining veins. Not every low-signal focus represents a true flow void. Rounded or punctate low-signal foci may represent phleboliths, calcification, thrombus, fibrosis, or hemosiderin deposition. In contrast, true high-flow vascular voids are typically tubular or serpiginous and should be corroborated by concordant findings on dynamic imaging. Time-resolved MR angiography is particularly useful for demonstrating arteriovenous shunting and early venous filling [10, 12].

Slow-flow malformations lack convincing fast-flow features and early arteriovenous shunting. Their morphology varies by vascular component: venous malformations (VMs) often show gradual enhancement on dynamic contrast-enhanced T1-weighted imaging or MR angiography, whereas lymphatic malformations (LMs) typically contain nonenhancing cystic spaces with variable wall or septal enhancement [7, 10, 15].

### Step 2: Identify the dominant vascular component

After assessment of flow characteristics, the dominant vascular component should be identified. Slow-flow malformations may be predominantly venous, lymphatic, capillary, or combined, and each component has different imaging features and treatment implications. Associated findings include T2-hyperintense venous lakes and contiguous draining veins in VMs, and cystic lymphatic components, septations, and fluid–fluid levels in LMs. Capillary malformations (CMs) are typically superficial and may show little or no discrete abnormality on MRI unless accompanied by soft-tissue overgrowth or other vascular components. When more than one component is present, the report should identify the predominant component and separately describe minor components rather than assigning only a combined-malformation label. This distinction is clinically relevant because the dominant vascular component may inform treatment planning, together with symptoms, lesion location and extent, venous drainage, functional impairment, prior treatment, and multidisciplinary assessment.

### Step 3: Assess tissue composition

The internal composition of the lesion should be assessed for fat, fibrosis, phleboliths, thrombus, hemorrhage, and hemosiderin deposition, as these findings may aid lesion characterization and differential diagnosis. Tissue composition should be interpreted together with vascular morphology. Dilated venous spaces, particularly when accompanied by phleboliths or thrombi, suggest a VM. Small amounts of fat may also be present at the periphery of or interspersed within the lesion. Multiloculated cystic spaces containing hemorrhagic or proteinaceous material may suggest a LM, particularly when associated with fluid–fluid levels, although these findings are not specific. In contrast, diffuse intramuscular fibrofatty replacement, low-signal fibrosis, and muscle atrophy should raise suspicion for FAVA.

### Step 4: Define lesion extent, anatomic relationships, and associated anomalies

A practical imaging report should clearly describe the full extent of the lesion and its relationship to adjacent structures, including the skin, subcutaneous tissue, muscles, bones, joints, neurovascular bundles, and named vessels. Relevant features include whether the lesion is intramuscular or intermuscular; the presence of intraosseous or intra-articular extension; its relationship to major vessels and nerves; the course of draining veins; communication with the deep venous system; and associated lymphatic edema or venous stasis [7, 10, 15]. Associated soft-tissue or osseous overgrowth, muscle atrophy or fatty replacement, limb asymmetry, and findings suggestive of an underlying syndrome should also be evaluated when present. These findings should be summarized in the report, with particular emphasis on features that may alter procedural access or increase treatment risk. A practical reporting checklist is provided in Table 3.

**Table 3:** Key MRI assessment points for vascular malformations in the 2025 ISSVA framework

Category	Key MRI assessment	Clinical relevance
Flow characteristics	Flow voids, enlarged feeding arteries, early arterial enhancement, early venous filling, arteriovenous shunting, delayed or progressive enhancement	Distinguishes fast-flow from slow-flow malformations and provides information relevant to treatment planning
Dominant vascular component	Venous spaces, dilated venous channels, lymphatic cysts, capillary component, septations, fluid–fluid levels, combined components	Supports classification as VM, LM, CM, combined malformation, or other entities
Tissue composition and associated changes	Fat, fibrosis, phleboliths, thrombus, hemorrhage, hemosiderin	Helps differentiate VM, LM, and FAVA
Lesion extent and anatomic relationships	Involvement of skin, subcutaneous tissue, muscle, bone, joint, neurovascular structures, relationship to named vessels or nerves, lymphatic edema, muscle atrophy, and osseous or soft-tissue overgrowth	Defines lesion extent and provides information relevant to syndromic assessment and procedural risk

*CM* capillary malformation, *FAVA* fibroadipose vascular anomaly, *LM* lymphatic malformation, *VM* venous malformation

### Fast-flow vascular malformations

#### Arteriovenous malformations and arteriovenous fistulas

**Fig. 3:**
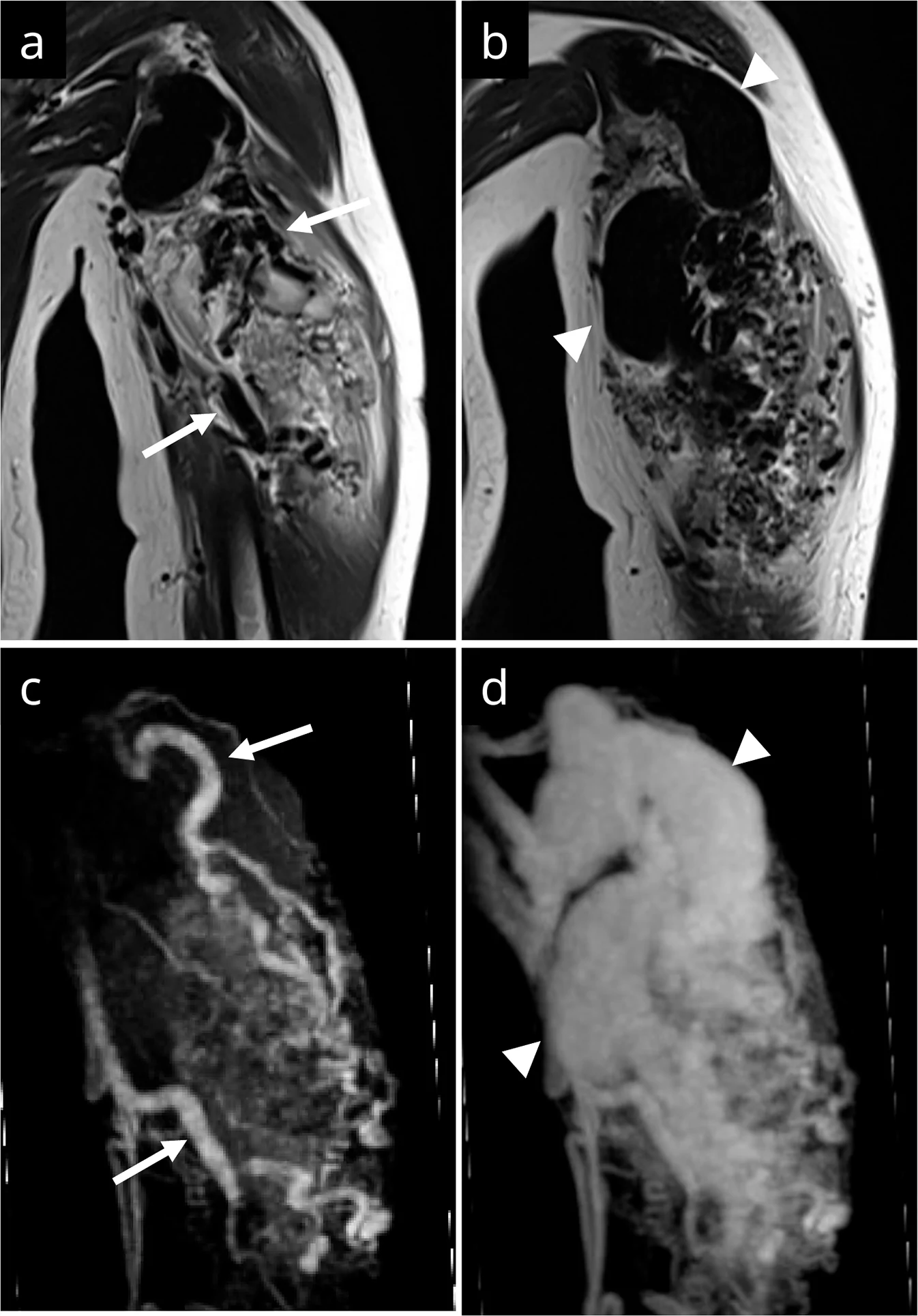
Arteriovenous malformation of the left upper arm in a 47-year-old woman presenting with interval enlargement, pain, and ulceration. **a**, **c** Fat-suppressed T2-weighted image (**a**) and arterial-phase 3D MR angiography (**c**) demonstrate multiple dilated and tortuous feeding arteries arising from branches of the brachial artery (arrows). **b**, **d** Fat-suppressed T2-weighted image (**b**) and venous-phase 3D MR angiography (**d**) show markedly dilated draining veins (arrowheads). The draining veins appear as signal voids, reflecting high-velocity flow. The arteriovenous shunting region appears as an ill-defined area with mixed high- and low-signal intensity on fat-suppressed T2-weighted images (asterisks). The combination of enlarged feeding arteries, a complex arteriovenous shunting region, and enlarged draining veins confirms fast-flow hemodynamics and defines the treatment-relevant angioarchitecture. The diagnosis was based on characteristic MRI/MRA findings and was confirmed by digital subtraction angiography

Arteriovenous malformations (AVMs) and arteriovenous fistulas (AVFs) are representative fast-flow vascular malformations. Somatic variants involving the RAS/MAPK pathway have been identified in extracranial AVMs and related fast-flow vascular anomalies [16]. AVMs typically show serpiginous flow voids, enlarged feeding arteries, early arterial enhancement, and early venous drainage, whereas AVFs show a direct arteriovenous communication without an intervening nidus. Time-resolved MR angiography is useful for identifying the feeding arteries, nidus or fistulous point, and draining veins (Fig. 3). In clinical practice, AVMs may also be characterized using the Schobinger classification for clinical severity and the Cho–Do classification for angiographic architecture and treatment planning [17, 18]. Although these classifications are not MRI-based systems, imaging helps define lesion extent, angioarchitecture, tissue involvement, and complications that are relevant to embolization, surgery, or combined treatment.

The 2025 ISSVA classification recognizes intramuscular fast-flow vascular anomaly (IFVA) as an isolated fast-flow vascular malformation. The accompanying ISSVA glossary introduces IFVA as a new term substituting for the former designation “intramuscular capillary-type hemangioma” [1, 2]. This terminology change reflects emerging clinicopathologic and molecular evidence, including the identification of somatic MAP2K1 or KRAS variants [19]. Unlike a typical AVM, IFVA may demonstrate prominent intramuscular vascularity and fast-flow features without a discrete nidus or unequivocal early venous drainage [20–22]. Because standardized imaging criteria remain limited and its imaging findings may overlap with those of other hypervascular intramuscular lesions, diagnosis requires integration of clinical, imaging, pathologic, and, when available, molecular findings [19–22].

Parkes–Weber syndrome is a congenital vascular disorder characterized by CMs and arteriovenous shunts, accompanied by overgrowth of the affected bone and/or soft tissue [1, 23]. It is classified as a syndromic fast-flow vascular malformation. Regarding terminology, the 2025 ISSVA glossary states that the term “Klippel–Trenaunay–Weber syndrome” is incorrect and should not be used because it conflates two distinct disorders: Klippel–Trenaunay syndrome and Parkes–Weber syndrome.

### Slow-flow vascular malformations

#### Venous malformations

##### Conventional venous malformation

In the 2025 ISSVA classification, VMs are categorized within the slow-flow branch and further organized into isolated, multifocal, and syndromic forms [1].

**Fig. 4:**
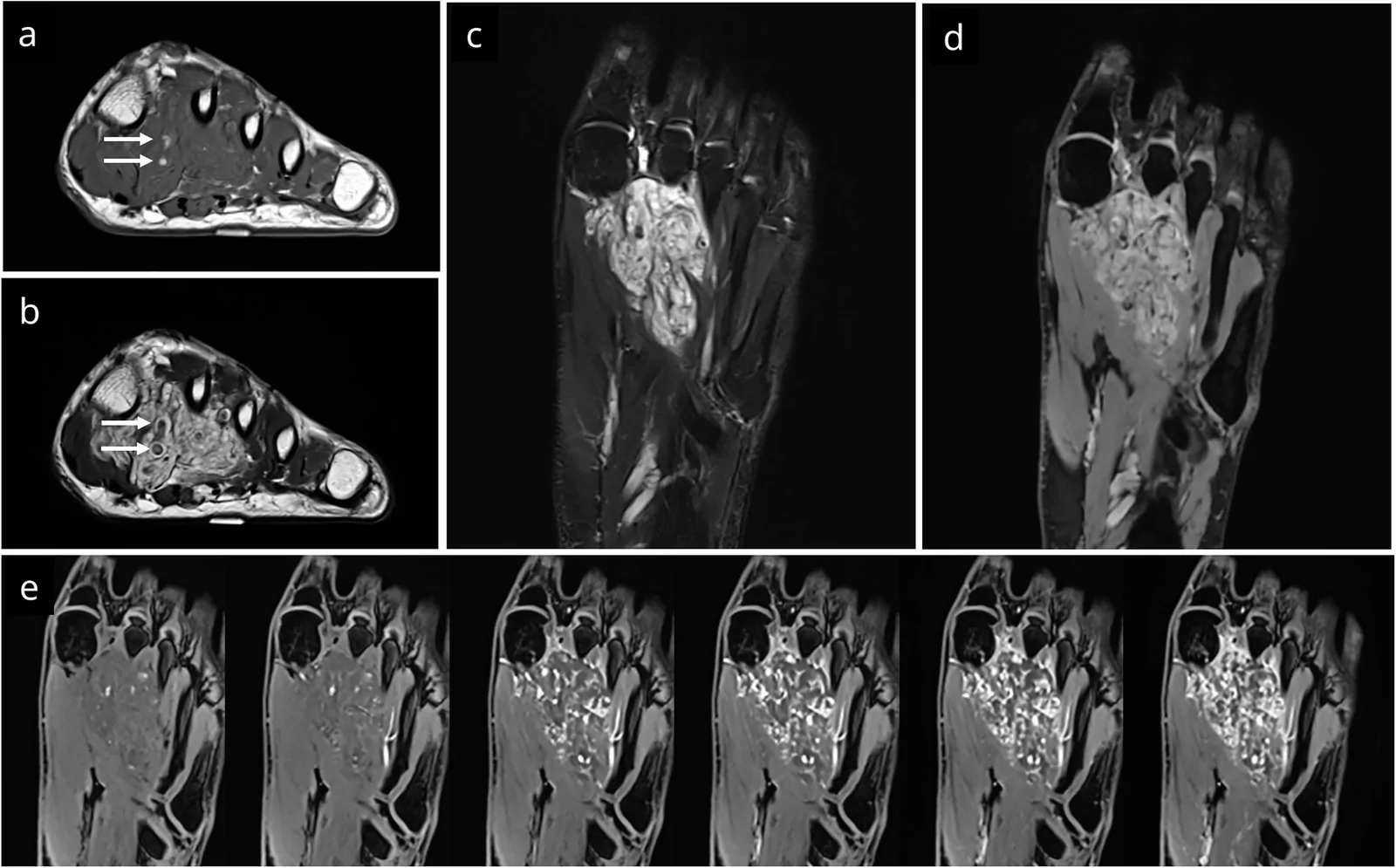
Venous malformation of the left foot in a 28-year-old woman. **a** Axial T1-weighted image shows an ill-defined lesion that is isointense relative to the muscle. **b**, **c** Axial T2-weighted image (**b**) and coronal fat-suppressed T2-weighted image (**c**) show mildly heterogeneous hyperintensity, consistent with predominantly venous spaces. **d** Coronal contrast-enhanced fat-suppressed T1-weighted image demonstrates heterogeneous enhancement. **e** Dynamic contrast-enhanced T1-weighted images demonstrate gradual heterogeneous enhancement, supporting slow-flow hemodynamics. Punctate foci that are hyperintense on the T1-weighted image (**a**) and hypointense on the T2-weighted image (**b**) are suggestive of thrombi (arrows in **a**, **b**). Although phleboliths may show a similar punctate appearance, no calcified foci were identified on plain radiographs (not shown). The diagnosis was based on the characteristic imaging findings, without histopathologic or genetic confirmation

VMs are congenital lesions that may become clinically apparent later in life and may cause pain, swelling, or functional limitation [15]. On MRI, they typically appear as well- or ill-defined lesions containing spongiform T2-hyperintense spaces or dilated venous channels with delayed or progressive enhancement (Fig. 4). The extent of involvement and venous drainage should be assessed because these features may influence lesion characterization, procedural planning, treatment selection, and response to sclerotherapy [7, 10, 15, 24, 25].

**Fig. 5:**
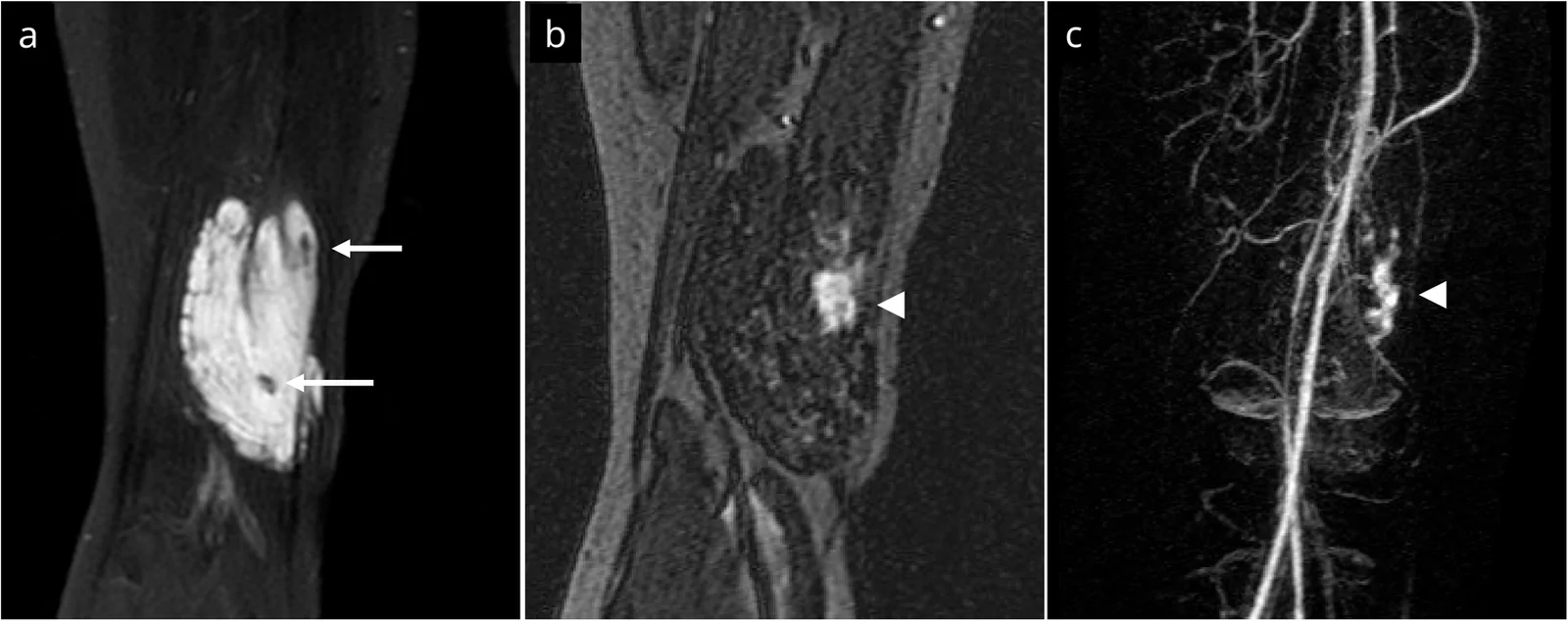
Venous malformation with focal early arterial-phase enhancement in the right leg of a 4-year-old boy. **a** Coronal fat-suppressed T2-weighted image shows an ill-defined hyperintense lesion containing punctate hypointense foci, which may represent phleboliths or thrombi (arrows). **b**, **c** Source image (**b**) and maximum-intensity-projection image (**c**) from arterial-phase three-dimensional MR angiography demonstrate focal early enhancement within the lesion (arrowheads). However, no definite nidus, fistulous connection, or early venous drainage is identified. The focal early enhancement should therefore be reported as an atypical hemodynamic feature rather than interpreted as definite arteriovenous shunting. The diagnosis was based on the characteristic imaging findings, without histopathologic or genetic confirmation

Although VMs are classified as slow-flow lesions, some lesions may demonstrate focal early arterial-phase enhancement or other atypical hemodynamic features (Fig. 5). Because these findings may influence treatment planning, they should be clearly documented in the imaging report.

##### Fibroadipose vascular anomaly

The 2025 ISSVA classification categorizes fibroadipose vascular anomaly (FAVA) as a slow-flow vascular malformation. FAVA should not be equated with a conventional intramuscular VM because its clinical and imaging features are characterized by tissue-dominant abnormalities rather than predominantly ectatic venous spaces [2, 26–28]. FAVA commonly affects the lower-extremity muscles in children and young adults and may cause persistent pain, contractures, and functional limitations [27, 29]. Somatic activating PIK3CA variants have been identified in a substantial subset of FAVAs, supporting their relationship to the PIK3CA-related vascular malformation spectrum [30].

**Fig. 6:**
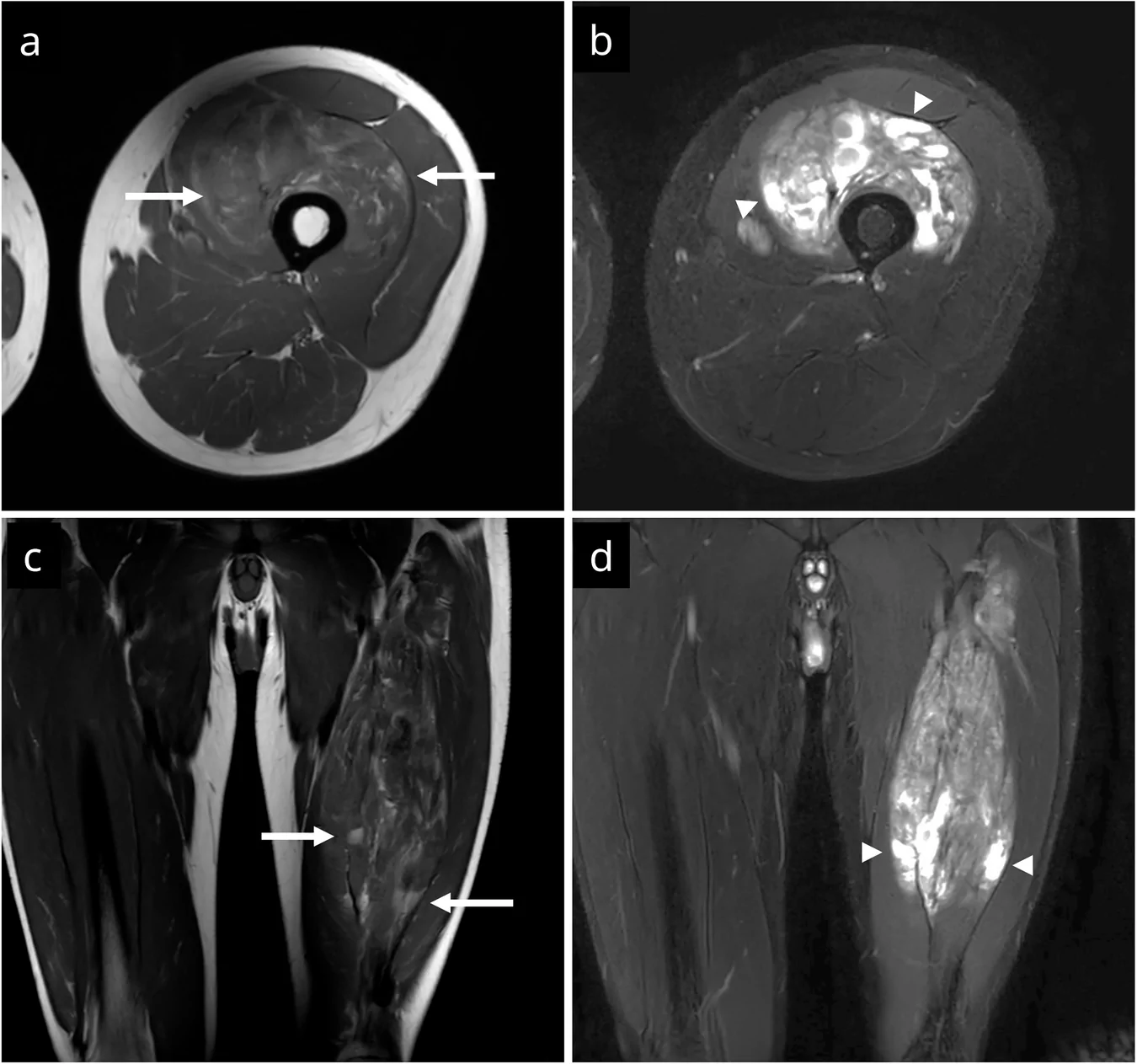
Fibroadipose vascular anomaly of the left thigh in a 22-year-old man presenting with persistent pain. **a**, **c** Axial and coronal T1-weighted images show an ill-defined heterogeneous intramuscular lesion containing T1-hyperintense fibrofatty components (arrows). **b**, **d** Axial (**b**) and coronal (**d**) fat-suppressed T2-weighted images demonstrate heterogeneous T2-hyperintense ectatic vascular channels (arrowheads) interspersed with areas of lower signal intensity corresponding to fibrous tissue. The combination of fibrofatty replacement, fibrosis, and ectatic slow-flow vascular components favors fibroadipose vascular anomaly rather than a conventional intramuscular venous malformation. The diagnosis was histopathologically confirmed. The lesion involves a long segment of the thigh musculature, a finding relevant to treatment planning

MRI typically demonstrates intramuscular fibrofatty replacement, fibrosis, muscle atrophy, and variable ectatic venous or lymphatic components (Fig. 6) [26–29]. Treatment is individualized according to lesion extent, symptoms, and involvement of adjacent structures [27, 31, 32].

Radiologists should report the extent of fibrofatty replacement and fibrosis, muscle involvement and atrophy, ectatic vascular components, and involvement of adjacent neurovascular structures. Characterizing the relative contributions of vascular, fibrofatty, and fibrous components may provide useful information for treatment planning. However, treatment selection should be individualized according to symptoms, lesion extent and location, functional impairment, prior treatment, and multidisciplinary assessment. The key differences between conventional VM and FAVA are summarized in Table 4.

**Table 4:** Key clinical, imaging, and treatment differences between VM and FAVA

Feature	VM	FAVA
Clinical presentation	Present at birth but may become clinically apparent later in life; may be asymptomatic or present with pain, swelling, or functional limitation	Usually affects children and young adults; persistent pain, contracture, and functional limitation are common
Location	Subcutaneous tissue or muscle; may extend across tissue planes and involve bone	Predominantly intramuscular, commonly involving the lower extremities
MRI features	Well-defined or ill-defined spongiform T2-hyperintense venous spaces or dilated venous channels, with delayed or progressive enhancement	Ill-defined intramuscular fibrofatty replacement, T2-hypointense fibrous components, muscle atrophy, and T2-hyperintense ectatic venous or lymphatic channels
Treatment considerations	Sclerotherapy is commonly used for symptomatic ectatic venous components; treatment planning should consider lesion extent, venous drainage, symptoms, and clinical context.	Treatment should be individualized according to symptoms, lesion extent, functional impairment, and the relative vascular, fibrofatty, and fibrous components; sclerotherapy, surgical excision, cryoablation, or targeted medical therapy may be considered in selected patients.

*FAVA* fibroadipose vascular anomaly, *LM* lymphatic malformation, *VM* venous malformation

#### Lymphatic malformations

**Fig. 7:**
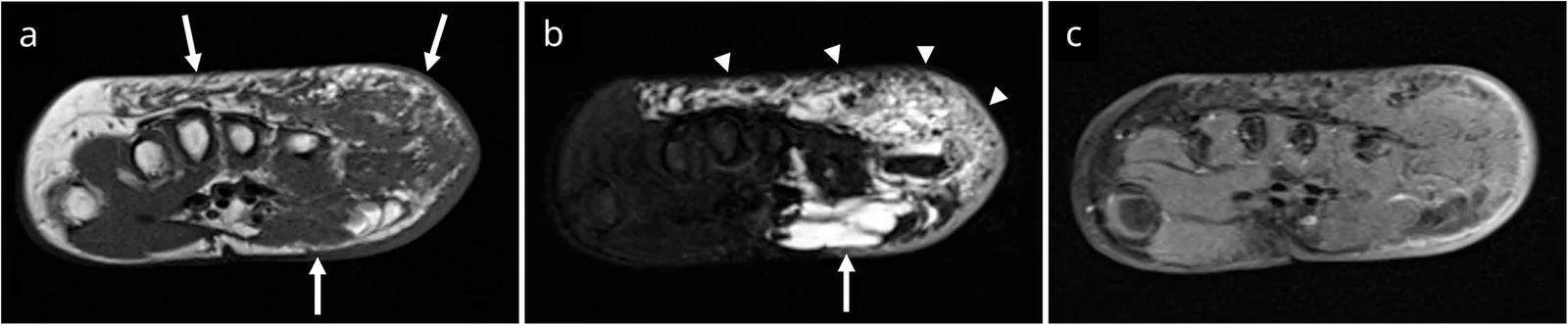
Mixed macrocystic–microcystic lymphatic malformation of the left hand in a 5-year-old boy. **a** Axial T1-weighted image shows an ill-defined lesion in the subcutaneous tissue (arrows). **b** Axial fat-suppressed T2-weighted image demonstrates a multiloculated cystic lesion with predominantly macrocystic components on the palmar aspect (arrow) and predominantly microcystic components on the dorsal aspect (arrowheads). **c** Axial contrast-enhanced fat-suppressed T1-weighted image shows only minimal thin septal enhancement, without substantial enhancement of the cystic spaces. The report should describe the relative distribution of the macrocystic and microcystic components and the extent of involvement because these findings may influence treatment selection and response to sclerotherapy. The diagnosis was based on the characteristic clinical course and imaging findings, without histopathologic or genetic confirmation

In the 2025 ISSVA classification, LMs are organized into isolated LMs, complex lymphatic anomalies, and lymphedema. Isolated LMs are further classified as macrocystic, microcystic, or mixed macrocystic–microcystic lesions (Fig. 7) [1]. Complex lymphatic anomalies comprise a heterogeneous group of disorders, including generalized lymphatic anomaly, kaposiform lymphangiomatosis, Gorham–Stout disease, and central conducting lymphatic anomaly, and may involve multiple organs and the central lymphatic system. Their evaluation may require whole-body imaging and dedicated lymphatic imaging techniques, including dynamic contrast-enhanced MR lymphangiography, in addition to conventional MRI [33].

**Fig. 8:**
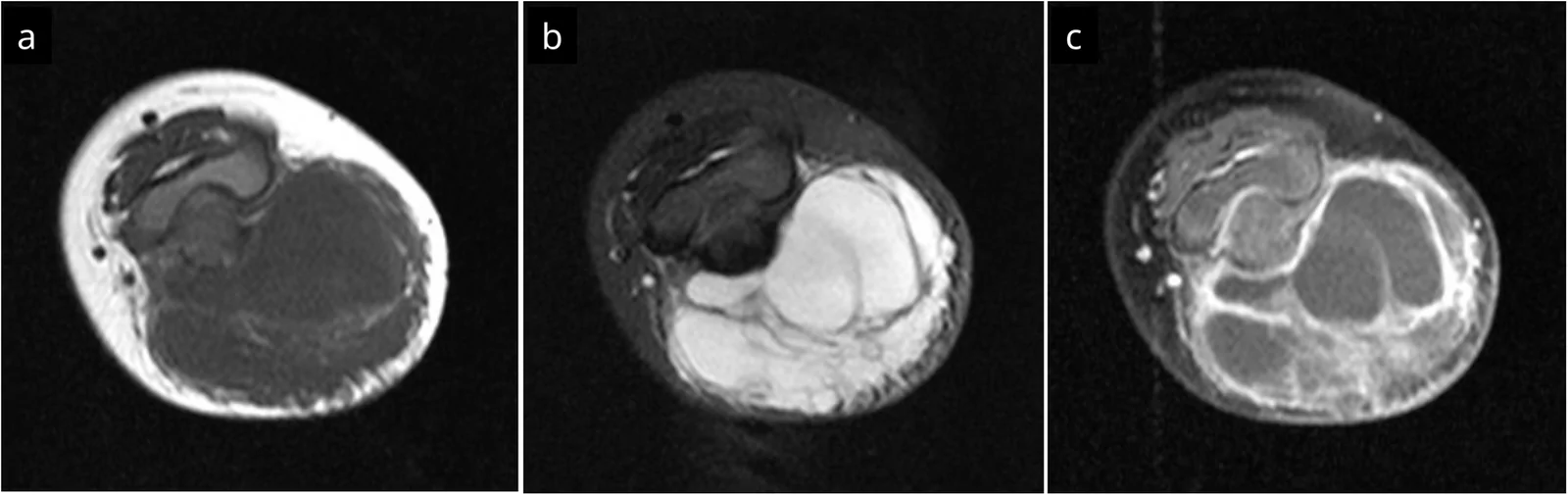
Lymphatic malformation with superimposed inflammation of the left arm in a 9-month-old girl. **a** Axial T1-weighted image shows an ill-defined mass lesion in the subcutaneous tissue. **b** Axial fat-suppressed T2-weighted image demonstrates a multiloculated cystic lesion with irregularly thickened walls and septa. **c** Axial contrast-enhanced fat-suppressed T1-weighted image shows avid enhancement of the thickened walls and septa. In comparison with an uncomplicated lymphatic malformation, irregular wall and septal thickening with marked enhancement suggests superimposed inflammation. The diagnosis was based on the characteristic clinical course and imaging findings, without histopathologic or genetic confirmation

Imaging assessment should include cyst size and distribution, septations, internal hemorrhage or proteinaceous content, and the pattern of wall or septal enhancement. Thin septal enhancement may be observed, whereas irregular wall or septal thickening with avid enhancement should raise concern for superimposed inflammation (Fig. 8) [15, 34]. Fluid–fluid levels are common, particularly in macrocystic or mixed lesions, and usually reflect hemorrhage or proteinaceous content. Macrocystic LMs generally respond more favorably to sclerotherapy than predominantly microcystic lesions [35–37].

Radiologists should report whether the lesion is predominantly macrocystic, microcystic, or mixed; document hemorrhagic or proteinaceous contents; and identify findings suggestive of superimposed inflammation, as these features may influence treatment selection and response.

#### Capillary malformations

Capillary malformations (CMs) may be inconspicuous on MRI and should therefore be evaluated in conjunction with clinical skin findings. In the 2025 classification, CMs are categorized by their clinical and cutaneous patterns, including port-wine, reticulate or telangiectatic, and geographic-pattern CMs, as well as their associated syndromic conditions [1]. This revision provides a clearer and more clinically intuitive framework.

**Fig. 9:**
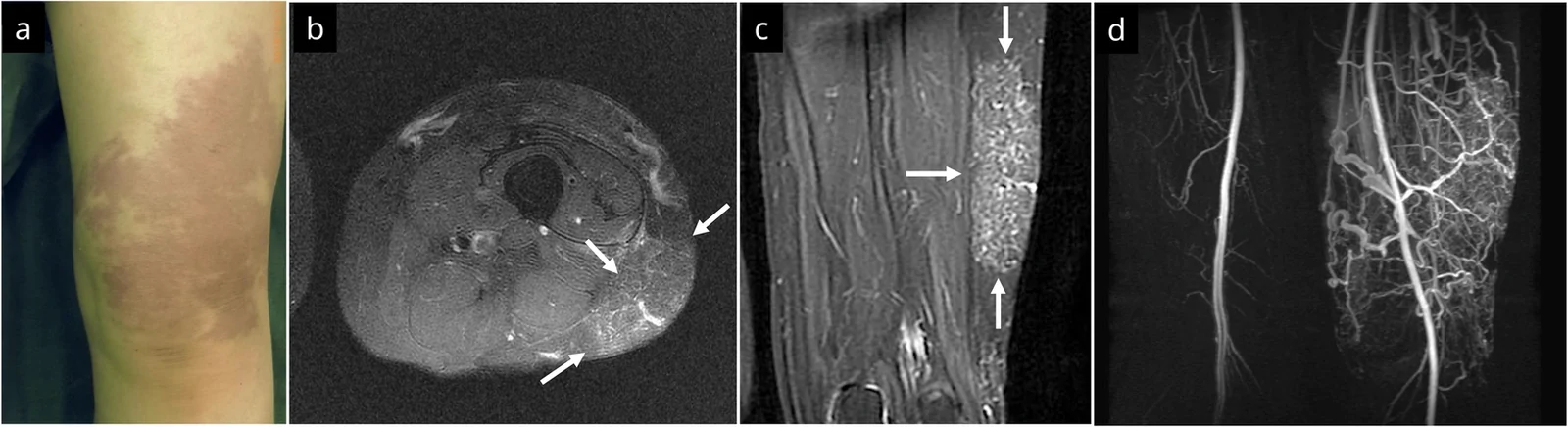
Presumptive low-resistance capillary malformation of the left leg in a 63-year-old woman presenting with persistent localized pain. **a** Clinical photograph shows a geographic dark-red capillary stain around the knee. **b**, **c** Axial (**b**) and coronal (**c**) fat-suppressed T2-weighted images demonstrate subcutaneous soft-tissue thickening and a cluster of fine hyperintense vascular structures corresponding to the cutaneous lesion. **d** Arterial-phase three-dimensional MR angiography shows prominent superficial vascular structures without a discrete nidus or enlarged feeding artery. Review of the complete dynamic series demonstrates no unequivocal early venous drainage. These findings indicate increased vascularity without characteristic arteriovenous shunting. Based on the characteristic cutaneous and imaging findings, a presumptive diagnosis of low-resistance capillary malformation was made; no histopathologic or genetic confirmation was available

The 2025 classification specifically recognizes low-resistance CM, also termed CM with faster flow, as a capillary-malformation subtype [1, 2]. This newly introduced category remains incompletely characterized in the imaging literature. These lesions may show increased vascularity or relatively early enhancement. However, in the absence of a demonstrable nidus, enlarged feeding arteries, or unequivocal early venous drainage, they should not automatically be classified as AVMs (Fig. 9) [1, 2]. To avoid overdiagnosis as a fast-flow malformation, radiologists should carefully assess the overall vascular architecture [1, 7, 10, 38]. Conversely, the presence of a demonstrable nidus, clear early venous drainage, or a defined arteriovenous shunt should prompt consideration of an associated fast-flow lesion, including CM–AVM syndrome in the appropriate clinical context.

#### Combined slow-flow malformations

In the 2025 ISSVA classification, combined malformations containing two or more slow-flow vascular components are categorized within the slow-flow branch. Examples include capillary–venous malformation, lymphatic–venous malformation, and capillary–lymphatic–venous malformation [1].

The concept of combined malformations containing multiple vascular components was already established in the previous classification. The principal change is organizational: flow characteristics are considered first, followed by identification of the individual capillary, lymphatic, and venous components. These lesions may occur as localized malformations or as part of syndromic conditions, such as Klippel–Trénaunay syndrome, which is classified as a capillary–lymphatic–venous malformation with limb overgrowth (Fig. 2) [1, 39].

Radiologists should avoid reporting only a broad combined-malformation or syndromic label. The report should separately describe the individual vascular components, their relative extent, flow characteristics, associated tissue or osseous overgrowth, developmental anomalies of named vessels, and treatment-relevant vascular connections.

### Developmental anomalies of named vessels

In the 2025 classification, malformations of major named vessels have been reclassified as developmental anomalies of named vessels [1]. The revised terminology emphasizes the developmental nature of these abnormalities and removes the implication that only major vessels are relevant.

Abnormalities of vessel origin, course, caliber, continuity, and communication were already recognized in the previous classification. The 2025 framework reorganizes these findings into a distinct developmental category that may coexist with fast-flow or slow-flow vascular malformations. A persistent lateral marginal vein typically courses along the lateral aspect of the lower extremity and may extend from the foot toward the pelvis. Its caliber, continuity, and communication with the deep venous system should be carefully assessed. In particular, when the deep venous system is hypoplastic or absent, the persistent lateral marginal vein may serve as an essential venous outflow pathway; therefore, adequate alternative venous drainage should be confirmed before surgical or endovascular occlusion [40, 41].

For radiologists, developmental anomalies of named vessels should be assessed separately from any accompanying vascular malformation. Reports should describe aplasia or hypoplasia, stenosis, dilatation, anomalous course or communication, persistence of embryonic vessels, patency of the deep venous system, and whether an anomalous vessel constitutes an essential drainage pathway. These findings may directly affect procedural feasibility and treatment-related risk.

### Pearls and pitfalls in imaging assessment

Several practical pitfalls should be considered when interpreting vascular malformations.

1. Signal voids should not automatically be interpreted as evidence of fast flow. Low-signal foci may also represent phleboliths, calcification, thrombus, fibrosis, or hemosiderin deposition. True high-flow vascular voids are typically tubular or serpiginous, and fast-flow characteristics should be confirmed by concordant findings, including enlarged feeding arteries, early venous filling, and characteristic vascular architecture on time-resolved imaging.
2. Focal early enhancement within a VM does not necessarily indicate arteriovenous shunting. Focal early enhancement should not be regarded as definite arteriovenous shunting in the absence of enlarged feeding arteries, early venous filling, or a demonstrable nidus or fistulous connection.
3. Intramuscular VM and FAVA should not be considered interchangeable. Although these entities may overlap on conventional MRI, predominantly ectatic venous spaces favor VM, whereas fibrofatty replacement of muscle and fibrosis favor FAVA.
4. Developmental anomalies of named vessels should not be overlooked. They should be evaluated separately from the accompanying vascular malformation. Their origin, course, caliber, continuity, and communication with the deep venous system should be described because these findings may influence classification, procedural planning, and treatment-related risk.
5. Combined malformations should not be summarized only by a broad diagnostic label. The individual vascular components, their flow characteristics, lesion extent, and treatment-relevant anatomy should be described separately.

## Discussion

The 2025 ISSVA classification provides an updated framework for organizing vascular anomalies, but its practical application in radiologic assessment requires translation of classification terminology into imaging-based observations. Rather than assigning a diagnostic label alone, radiologists can characterize vascular malformations according to flow characteristics, dominant vascular components, tissue composition, lesion extent, associated anomalies, and treatment-relevant anatomy. The 2025 ISSVA classification and the four-step workflow proposed in this review represent distinct levels of organization: the former is an established classification framework, whereas the latter is an author-derived educational framework that organizes established imaging observations into a practical sequence aligned with the terminology and structure of the 2025 ISSVA classification. The proposed workflow is not part of the official ISSVA classification and should not be interpreted as an ISSVA-endorsed diagnostic or reporting algorithm.

An important implication of the updated framework is that imaging reports should reflect not only the presumed malformation type but also the anatomical and hemodynamic features that may influence management. These include feeding arteries, draining veins, the presence of a nidus or fistulous connection, communication with the deep venous system, developmental anomalies of named vessels, tissue overgrowth, and involvement of adjacent structures. Such findings may provide clinically relevant information for treatment selection and assessment of procedural risk beyond the diagnostic category alone. Treatment decisions should therefore integrate imaging findings with symptoms, lesion location and extent, functional impairment, prior treatment, and multidisciplinary assessment rather than be based on imaging phenotype alone.

However, the ISSVA classification is not an exclusively imaging-based system. Some vascular anomalies cannot be classified confidently on imaging alone, and integration with clinical presentation, physical examination, histopathology, and molecular findings may be required. In addition, overlapping imaging features may occur among certain entities, particularly VMs, FAVA, and other complex intramuscular vascular anomalies. Radiologists should therefore avoid overconfident categorization when characteristic features are absent and should clearly describe the observed imaging findings.

The proposed workflow is intended as a practical educational framework rather than a validated diagnostic algorithm. Its diagnostic performance, reproducibility, and interobserver agreement have not been formally evaluated. Further studies are needed to validate imaging criteria for recently recognized or reorganized entities and to determine how structured imaging assessment affects diagnostic agreement, treatment selection, and clinical outcomes. Future studies are also needed to determine whether standardized imaging assessment and reporting provide measurable benefits in clinical practice.

## Conclusion

The 2025 ISSVA classification provides an updated organizational framework for vascular anomalies, including a flow-based structure for vascular malformations. In this educational review, we propose an author-derived four-step framework for organizing established MRI observations of peripheral and soft-tissue vascular malformations, including hemodynamic characteristics, vascular components, tissue composition, lesion extent, and treatment-relevant anatomy. This framework is intended as an educational and organizational tool rather than a validated diagnostic algorithm, and its diagnostic, reporting, and clinical utility requires future evaluation.

## Data Availability

No datasets were generated or analyzed for this educational review.

## Abbreviations

AVF: Arteriovenous fistula
AVM: Arteriovenous malformation
CM: Capillary malformation
FAVA: Fibroadipose vascular anomaly
IFVA: Intramuscular fast-flow vascular anomaly
ISSVA: International Society for the Study of Vascular Anomalies
LM: Lymphatic malformation
MRI: Magnetic resonance imaging
PUVA: Potentially unique vascular anomaly
VM: Venous malformation
